# Impact of corporate social responsibility on employee loyalty: Mediating role of person-organization fit and employee trust

**DOI:** 10.1371/journal.pone.0300933

**Published:** 2024-03-21

**Authors:** Hebo Jin, Xuexiao Li, Guangsen Li

**Affiliations:** College of Management, Qingdao University of Technology, Linyi, PR China; University of Almeria: Universidad de Almeria, SPAIN

## Abstract

The study explores the impact of employee perceptions of corporate social responsibility (CSR) in improving employee loyalty by examining the direct and indirect role of person-organization fit and employee trust. A convenient sampling technique was employed to collect the data sample. A total of 338 questionnaires were collected at three different China-Pakistan Economic Corridor (CPEC) projects in Pakistan. The SmartPLS-3 was used to test the study hypotheses. The results revealed that CSR positively and significantly influenced employee loyalty. The findings indicated a partial mediating impact of P-O fit and employee trust in the relationship between CSR and employee loyalty. Discussions, implications, limitations, and future research direction are also given.

## 1. Introduction

Corporate social responsibility (CSR) has gained extensive consideration over the last three decades, and several empirical research has established a significant influence of CSR on employee performance [1, 2]. Organizational researchers have noticed the environmental concerns of project sectors that have faced the sustainability challenges such as air pollution and ground pollution [1]. Project employees are increasingly focused on adopting pro-environmental and pro-social practices. It is evident that different CSR initiatives enhance a company’s image and reputation, which promotes employee positive behavior [3]. For instance, a prior study found that CSR encourages employees’ emotional attachment to the corporation, leading to high performance [4]. Following this perception, scholars have examined the positive impact of CSR on employees’ job satisfaction [5], low turnout intention [4], and innovative behavior [6]. Therefore, it is crucial to identify the impact of employee perception of CSR reputation on their organizational loyalty.

Bowen [7] defines CSR as ’the responsibilities of a corporation to pursue those strategies, make those decisions or implement those action which is favorable to the society. A few studies have practically or conceptually examined the CSR-loyalty link in different settings such as Latif et al. [3] found a positive relationship between CSR and customers in the hotel industry, industrial sectors [8], and environmental perspectives [1]). Some studies also discovered its positive link to organizational psychology [9]. However, whether CSR activities affect employee loyalty in the project sectors still needs further consideration. Therefore, this study is the first attempt to examine the implications of CSR activities on employee loyalty in the China-Pakistan Economic Corridor (CPEC) project in Pakistan.

First, this research provides empirical evidence that emphasizes the importance of CSR activities for project organizations. Despite the extensive growth of business sectors, the previous study lacks to highlight how employee perception of CSR initiatives affects their loyalty in the project sectors [10]. In the management field, previous research indicates that corporate social responsibility shows a direct and indirect impact on a number of favorable outcomes, such as enhancements in performance quality [11], work satisfaction [12], and well-being [13]. Therefore, conceptual research that leans toward project organization contexts (e.g., CPEC projects) is needed. Second, the present study suggests a research model that investigates how a project sector’s CSR reputation impact person-organization fit (P-O fit) and employee trust. Companies’ CSR activities significantly impact employee awareness [5]. The company’s CSR activities are linked with employee recognition of CSR reputation and employee favorable reactions and attitudes [4]. However, the effectiveness of CSR activities on employees’ behavior can vary on the project nature.

Third, the mediating role of P-O fit and employee trust in the relationship between CSR and employee loyalty has not yet been investigated in an integrated way till now. The previous scholar has explored the role of a potential mediator between the CSR-loyalty link. For instance, Oo et al. [14] discussed the mediating impact of P-O fit between CSR and employee organizational behavior. Ahn et al. [15] observed the mediating role of trust in the service industry. Yet, no research has acknowledged the mediating role of P-O fit and trust as mediating factors in a solo research model in the context of the project sectors.

This study designed a conceptual model that incorporates P-O fit and employee trust as mediating variables between employee perceptions of CSR and employee loyalty in CPEC projects. By doing so, this study is based on the two theoretical approaches ((i.e., stakeholder and social identity theory). It enlarges prior research that has grounded on the perspective of social identity exclusively to comprehend the CSR link with loyalty. Finally, the data sample of employees working on CPEC projects provides a unique context to validate the outcomes of employee perceptions of CSR activities in different project sectors. This may help both scholars and practitioners to focus their CSR actions and investments better. The current study sets itself apart by extending earlier studies by including the mediation effects in the context of project employees.

The organized structure of this paper is as follows: the introduction is covered in the first section. The second part addresses a theoretical background and hypothesis development, and the next section explains the research methodology. Finally, the last section provides data analysis, results, research contribution, limitations, and future direction.

## 2. Theoretical background and hypothesis development

### 2.1 Corporate social responsibility

According to Bowen [7], "CSR is the obligation of an organization to follow those strategies, decisions, or to peruse those guidelines which are favorable for social values and welfare," Later on, the concept of CSR advanced from being an environmental strategy to a strategic business necessity that helps organizations to achieve a competitive edge more efficiently [16]. Consequently, today CSR is considered a business tactic to accomplish organizational goals [17]. Several studies also discovered a direct and indirect link between CSR and organizational performance (e.g., corporate image and brand value) [18, 19], while some study shows its positive impact on employee behavior [20]. Employee perception of CSR is linked to the organization’s evaluation.

In the organizational context, it has been found that projects practicing CSR activities are more effective in building a strong connection with employees. Latif and Sajjad [21] reported that the CSR-organization relationship is influenced by stakeholder theory. A stakeholder is a person, entity, or organization that runs the organizational task in order to accomplish strategic objectives [22]. Hence, bringing about stakeholders’ perceptions and expectations is imperative, as they can significantly influence corporate performance. In doing so, organizations need to link their corporate social responsibility initiatives to their stakeholder’s desires and implement CSR initiatives that are aligned with the business strategy [23].

This being so, previous scholars argued that CSR activities are linked with favorable outcomes in service organizations. However, the quality of these services deeply relies on the employees [18]. Hence, most organizations consistently put a high focus on CSR activities to satisfy their employee to retain them loyal [22, 24], which create a corporate image, customer satisfaction [5], and positive perception among stakeholders [3]. Although previous literature discovered a positive relationship between CSR and employee work behavior, yet, research in the CPEC context is lacking. A prior study reported that employee behavior, such as devotion to the organization and work commitment, is directly influenced by how employees perceive CSR efforts [25]. It has been found that CSR initiatives by organizations significantly enhance employee loyalty [25, 26]. Another study validates that CSR effort by organizations is directly associated with employee positive work attitude such as trust, well-being, and self-motivation, and thus employee becomes more loyal to the organization. This study focuses on employee loyalty based on CSR efforts by the project organization. CSR has been potentially proven applicable attribute in the context of project sectors. Employee loyalty no longer relies solely on bonuses and awards but also on welfare and goodwill at the moral and philanthropic levels, which can inspire employee work behavior [18].

### 2.2 CSR and employee loyalty

Organizational Strategic goals can be achieved when employees display higher loyalty. As a psychological intention, employee loyalty is defined as a feeling or emotional attachment and commitment to the organization [27]. Similarly, Cachón‐Rodríguez et al. [28] explain employee loyalty as a behavioral conduct of an employee to remain within the organization. In the project sectors, leaders try to inspire and retain employees by focusing on those aspects that significantly affect employees’ decision process. By doing so, project sectors need to initiate the most effective trends, such as workshops, training and development, transparency, and knowledge-sharing process, which help to cope with employee work pressure [29].

In the organizational context, the employee perspective of corporate initiatives in the area of corporate social responsibility is a potential research topic for project sectors to boost employee loyalty [20]. CSR is noticed as a major element that creates awareness and guides the behaviors of stakeholders, customers, and employees [19]. Consequently, CSR plays an influential role not solely in the business literature [21] but also among scholars and practitioners [3]. Scholars conclude that CSR carries a strategic significance on employee loyalty. For instance, corporate social responsibility activities facilitate employees to recognize the organization based on the positive feelings they create about it [18]. The positive assessments of an organization’s CSR activities reinforce employee loyalty. Similarly, Smith and Kumar [30] study revealed that the CSR activities of an organization are a key factor in motivating and attracting employees, which helps them to engage in job tasks more efficiently.

Moreover, social identity theory explains the CSR-loyalty relationship in the context of project sectors [31]. The theory indicates employee positive CSR perceptions develop a positive image which increases employees’ self-esteem and organizational commitment. In a similar aspect, Mao et al. [32] acknowledged the positive link between CSR and employee psychological attachment to the organization. For instance, prior research found the significance of CSR activities in different business sectors with several positive outcomes such as customer satisfaction [3, 21], improvements in service quality [33] (Hur et al., 2020), corporate image and reputation [3, 34], employee wellbeing [16] and employee satisfaction [20].

Employees display positive attitudes when they feel the organization contributes to their well-being. This is so when employees experience and perceive that the organization is initiating appropriate CSR activities; consequently, employee tendency toward the organization becomes higher and shows high commitment, including job satisfaction and organizational loyalty [1, 35]. Based on the social identity theory, prior studies have found a significant influence of CSR on employee positive work behavior (See Table 1). However, there is still a research gap exist which can be identifying the CSR-employee loyalty relationship in the context of CPEC projects. Hence, the contradicting findings from existing literature strengthen the necessity to evaluate the effect of employee perceptions of CSR on employee loyalty in the setting of a project organization.

H1. There is a positive impact of CSR on employee loyalty.

**Table 1:** CSR-employee loyalty link.

Authors/Years	Organizational Sectors	Country	Findings
[36]	Manufacturing and service firms	India	Shows a positive impact of CSR on employee attitude and organizational loyalty.
[13]	hotels industry	China	CSR initiatives increase increased employee well-being, work commitment, and green behaviors.
[5])	financial institution	Latvia	Employee perceptions of CSR are significantly related to standard performance and work engagement.
[16]	SMEs	Spain	The findings revealed a positive relationship between CSR and firms’ image and reputation, including employee job performance.
[37]	Hotel industry	Pakistan	A significant positive link between CSR and employee innovative work behavior was found.

### 2.3 CSR and P-O fit link

In this model, the CSR-employee loyalty link is indirectly influenced by the mediation of P-O fit and employee trust. Employee loyalty is significantly strengthened by organization CSR efforts such as employee-ethical issues, well-being concerns, and environment-related challenges [23]. The stakeholder theory enlightens that CSR efforts can create a positive image and reputation for an organization; consequently, employees perceive that their values match the organization [38]. Prior research revealed that when employees’ and organizations’ values are parallel, employees display more efficient work attitudes [39]. Scholars found that employee perception of P-O fit is the consequence of CSR efforts [1, 40]. Therefore, organization CSR activities are beneficial for companies by building a strong job fit perception among employees, which in turn, leads to high work commitment [38].

Moreover, as Duarte and Mouro [39] have stated, organizational values and characteristics are reflected in CSR policies and practices, and employee perceptions about CSR create a foundation for their assessment of P-O fit. Consequently, a positive link between CSR and P-O fit can be predicted. CSR can influence employee perception of resemblance or suitability toward the organization and enhance a sense of belongingness [14]. This relationship was confirmed by Rawshdeh et al. [41], who reported that employees’ perceived P-O fit is significant when organizations involve in CSR practices. Duarte and Mouro [39] assert that a strong CSR initiative can serve as a signal for favorable outcomes (e.g., satisfaction and loyalty), which specify significant factors of job fit (e.g., benevolence and integrity). Therefore, when employees find CSR-related efforts, they show high intention and consider themselves fit for that organization. Hence, we hypothesize.

H1a. CSR positively influences employee perception of P-O fit.

### 2.4 CSR and employee trust link

Prior studies have found a positive impact of CSR on employee trust [42]. When employees perceive that an organization’s CSR activities are aligned with their values and attributes, the trust between employees and the organization increases [43]. Employees’ assessment of trust is significantly linked to the organization’s CSR reputation [4]. Organizational CSR efforts can build employees’ trust and positive perceptions of self-belonging. According to the social exchange theory, CSR initiatives create employee trust within the organization through reciprocal exchanges [5]. Employees who perceive their organization to be more involved in CSR activities display high organizational citizenship behavior. Ahmad et al. [44] (2020) found that the CSR mechanism is a route by which organizations generate employee trust, leading toward favorable outcomes (e.g., work commitment, Low turnout rate, and job satisfaction). Research acknowledged that CSR is a significant contributor to creating and building employee trust in a service organization [5]. Therefore, in the CPEC project context, CSR initiatives can trigger employee trust. Accordingly, we hypothesize:

H1b. CSR positively influences employee trust.

### 2.5 P-O fit-employee loyalty link

Studies revealed that fit between employee and organization is correlated with various work attitudes, including work engagement and organizational loyalty [45]. P-O fit emphasizes how employee and organizational values align, and it may have an effect on work engagement. Ashfaq and Hamid [46] argued that when individual skills, abilities, and values match with those of the organization, it increases their work productivity and psychological attachments. Similarly, Holland’s [47] job fit theory concluded that employees experience greater job satisfaction and effectiveness when they perceive a good match between their skills and the value of an organization. As Kim and Gatling [48] found that employees’ lower perceptions of job fit cause absenteeism and high turnover; however, higher perceptions of job fit accelerate job satisfaction and organizational loyalty. Therefore, in the settings of CPEC project sectors, employees whose values are aligned with the organization can display high organizational loyalty. Therefore, this study proposes that:

H2: P-O fit influences employee loyalty toward CSR organizations.

### 2.6 Employee trust-loyalty link

Currently, project sectors focus their concern on employee retention. The research evidence revealed that employee retaining is more effective compared to hiring a new candidate. Recent organizational literature explains the influence of employee trust on their loyalty [49]. Scholar like Chams-Anturi et al. [50] conducted a study on employee trust and loyalty. They pointed out employee trust as a critical determinant of employee work commitment and organizational loyalty. Moreover, the literature revealed by Dai et al. [51] concluded that trust builds strong psychological attachments and guides employee work behavior toward organizational loyalty. In the context of business sectors, empirical research confirmed the impact of employee trust on a number of positive outcomes in different corporate sectors. For example, Helliwell and Huang [52] found that employee trust enhances job satisfaction, well-being, and innovative work behavior [49]. According to the social exchange theory, employees’ work behaviors and attitudes are influenced by the level of trust they perceive in the organization [51]. In addition, Kim and Park [53] noted that trust shapes employees’ attitudes and behaviors in the workplace. When trust is built between employees and the organization, they are more likely to reciprocate this trust and display positive work behavior, such as organizational loyalty. Employee trust is considered an antecedent construct of work behavior and a significant factor in employees’ loyalty [50].

Based on the above discussion, this study proposes that employee trust plays a significant role in developing loyalty toward the project sector. Therefore, we propose the following hypothesis:

H3. Employee trust influences employee loyalty toward CSR organizations.

### 2.7 Mediating role P-O fit and employee trust

However, scholars have argued various factors can strengthen the CSR-loyalty relationship. For instance, Latif et al. [3] reported that numerous variables could mediate the CSR-loyalty relationship. Yet, there is a limited understanding of the indirect impact, such as P-O fit and employee trust, which link employee perceptions of CSR to their loyalty in the CPEC projects. Previous research found the impact of P-O fit and employee trust on employee work behavior. Hu et al. [54] indicated that individual P-O fit values determine employee perception. These values can also be significant factors in an employee’s decision to choose an organization that engages in more CSR activities. For example, a study by Konte et al. [55] found that P-O fit mediated the CSR-loyalty link. They revealed that employees who perceived a strong alignment between the organization’s CSR initiatives and their own values were more likely to display high organizational loyalty. Similarly, in the context of service sectors, Farooq et al. [56] reported that trust plays a crucial role in establishing and maintaining long-term relationships between an individual and an organization. The theory of organizational behavior suggests that employee trust could serve as a significant mechanism by which CSR activities affect employees’ positive work attitudes and behaviors [57]. Loor-Zambrano et al. [58] argued that employee trust is the most relevant module that influences CSR efforts, which in turn, leads to organizational loyalty.

Therefore, in the CPEC project setting, P-O fit and employee trust may influence the link between CSR and employee loyalty. Consequently, we hypothesize:

H4: P-O fit and employee trust mediate the relationship between CSR and employee loyalty.

**Fig 1:**
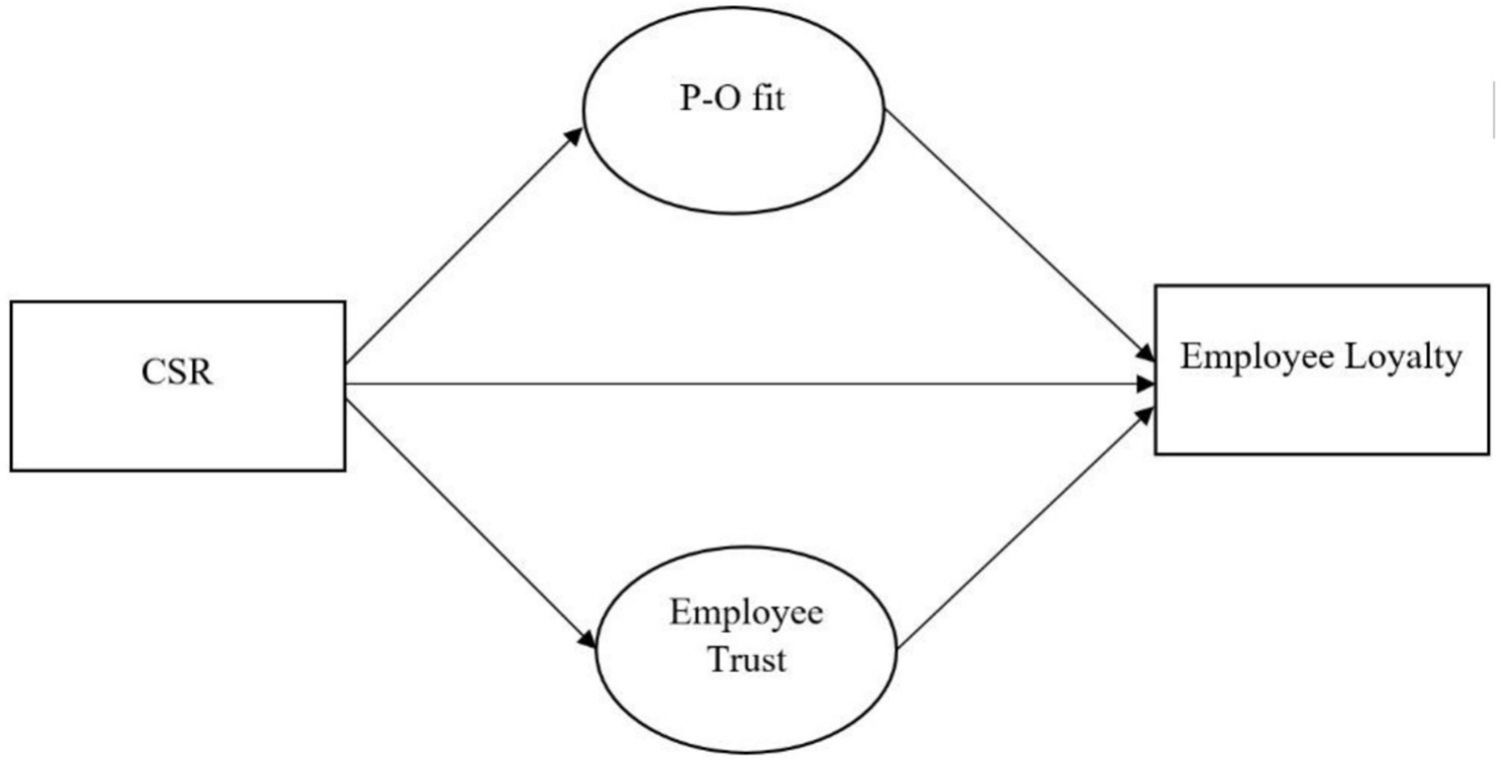
Conceptual framework.

The present study suggests the direct impact of CSR on loyalty H1, P-O fit H1a, and employee trust H1b. H2 indicates the impact of P-O fit on loyalty, while H3 shows the impact of employee trust on loyalty. H4 analyzes the indirect impact of P-O fit and employee trust on the relationship between CSR and employee loyalty (Fig 1).

## 3. Research methodology

### 3.1 Ethics approval

We, the authors of the research article mentioned above, want to clarify that we have no financial or non-financial interests that could potentially affect the honesty, fairness, or impartiality of the research presented in this article. Additionally, there are no personal or professional associations that might create a conflict of interest in relation to this study. Our research was conducted with honesty, transparency, and in line with ethical standards. We have taken all necessary steps to ensure the accuracy and reliability of the data and results presented in this article Academic Development and Ethics Committee Reference No. 2023/USTB/212. In this research, ethical standards were rigorously upheld. All participants provided informed consent, and a written consent statement was obtained from each individual involved in the study. This commitment to informed and documented consent ensured that participants were fully aware of the research’s objectives and their participation, thereby safeguarding their rights and privacy throughout the research process.

### 3.2 Data collection

The data sample consisted of three CPEC projects operating in Pakistan, which include power generation, Infrastructure, and Railway projects. Using a convenience sampling technique, we employed a survey methodology by developing a questionnaire. This method was used due to its established credibility and reliable research technique commonly used by scholars. This method is appropriate when obtaining a complete sampling frame. This type of sampling approach is well-suited for enabling theoretical generalization of the outcomes. The questionnaire survey method is cost-effective and suitable for contacting small and large groups, allowing the quick collection of data samples for statistical analysis [59]. From March 2023 to April 2023, a total of 450 questionnaires were distributed, with 150 questionnaires in each project. A total of 351 questionnaires were collected, where 338 were validly considered for further statistical analysis (response rate = 75.1%). The rest were neglected due to the missing data. Demographically, there are 251 male respondents (74.2%) and 87 female respondents (25.8%) as shown in Table 2.

**Table 2:** Sample characteristics.

Variable	N = 338	%
Gender	Male	74.2
	Female	25.8
Age	18–25	19.6
	26–30	34.3
	Above 30	46.1
Experience	Less than 2 years	22.4
	2–5	30
	Above 6	47.6
Project Sectors	Power generation	30.4
	Infrastructure	43.8
	Railway	25.8

Considering PLS-SEM guidelines, the data sample satisfies the required minimum criteria suggested by Westland [60]. Our model is based on four latent constructs and 24 indicators. The commonly used criterion for determining the minimum sample size is 10 responses for each indicator. In this research model, a total of five indications point to the construct; therefore, our sample of 338 meets the sampling adequacy criteria [60]. Demographic questions, such as age, gender, and region, were included in the first section of the survey. The second section contained questions to measure how employees perceive CSR, P-O fit, employee trust, and employee loyalty.

### 3.3 Measurements

For all the measurement items, a 5-point Likert scale was used to evaluate the items, with 1 denoting “strongly disagree” and 5 denoting “strongly agree”. A total of 8 items for employee perception of CSR were taken from the prior study [61, 62]. However, these item scales were conducted for other service industries (Hotels and Restaurants). Therefore, the item scales were reworded to fit the context of the study. The P-O fit has been measured with 6 items developed by Afsar and Badir [63]. The items include (e.g., “My personal values are aligned with my organization’s values and culture.”). For employee trust, 4 items were considered that were by [64]; hence the item scales were rearranged according to the present study, for example, “I believe that I can get organizational support and help if I needed”. Finally, employee loyalty was operationalized using 6 items adopted from Latif et al. [3] and Ineson et al. [65], the item scale includes “I will happily continue working for this organization in the future”.

## 4. Data analysis and results

### 4.1 Common method bias (CMB)

The **Common method bias** was assessed through the variance inflation factor (VIF) values of the inner model. In the current study, all the VIF values are lower than 3.33, the model can be considered free from common method bias [66].

### 4.2 Reliability and validity analysis

The authors used the SEM technique by running SmartPLS-3.3. The instruments were initially evaluated for validity and reliability. Next, SEM was run to test the conceptual proposed hypotheses. Gudergan et al. [67] suggest that Smart PLS 3.3 path modeling shows a substantiated technique for estimating simple and complex cause-and-effect model estimation in management science research. PLS-SEM can easily analyze a maximum number of variables, items, and structural relationships [68].

**Fig 2:**
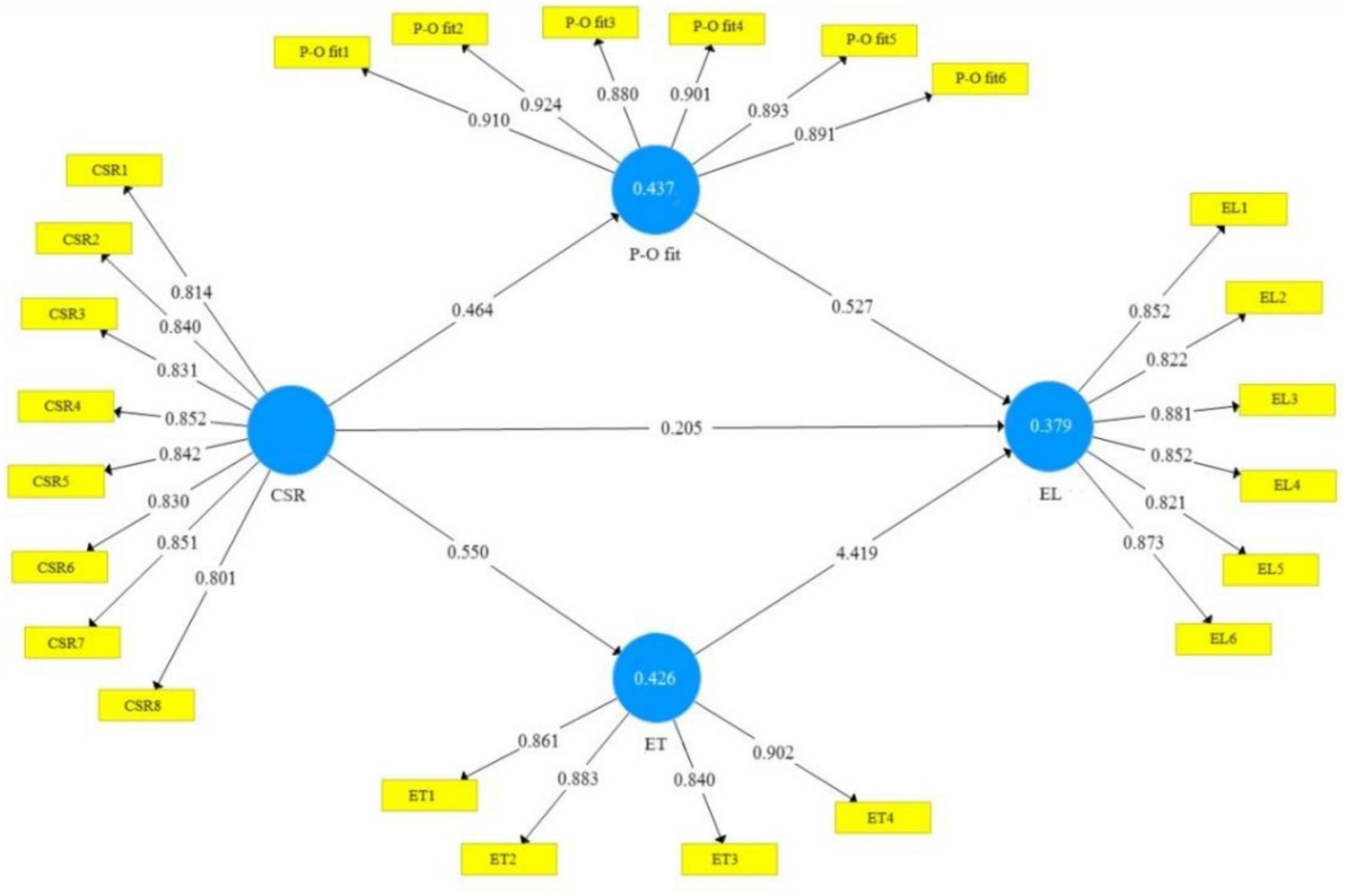
Measurement model.

The measurement model was run to assess the reliability and validity of the construct. Initially, factor loading values were assessed, items with factor loadings below 0.5 must be excluded only if their removal increases the recommended criteria of composite reliability (CR) and average extracted variance (AVE) [68]; therefore, three times scale were deleted which do not meet the minimum criteria of 0.5 (see Fig 2). The remaining factor loading exceeded the standard criteria of 0.5 suggested by Hair et al. [68], as shown in Table 3. The alpha values of all constructs meet the standard value of 0.7. CRs value of all constructs exceeded the criteria of 0.7. The AVE values of the overall construct show a higher range of 0.500, which suggests good reliability [69]. By measuring the correlations between the latent variables and the square root of AVE, discriminant validity was evaluated [70], and the HTMT ratio of correlation shows a threshold value below 0.85, as reported in Table 4.

**Table 3:** Item loadings, reliability, and validity.

CSR	Factor loading	Alpha	CR	AVE
CSR1	0.81	0.88	0.91	0.69
CSR2	0.84			
CSR3	0.83			
CSR4	0.85			
CSR5	0.84			
CSR6	0.83			
CSR7	0.85			
CSR8	0.80			
**P-O fit**				
P-O fit1	0.91	0.89	0.92	0.67
P-O fit2	0.92			
P-O fit3	0.88			
P-O fit4	0.90			
P-O fit5	0.89			
P-O fit6	0.89			
**Employee Trust**				
ET1	0.86	0.86	0.89	0.75
ET2	0.88			
ET3	0.84			
ET4	0.90			
**Employee Loyalty**				
EL1	0.85	0.85	0.89	0.72
EL2	0.82			
EL3	0.88			
EL4	0.85			
EL5	0.82			
EL6	0.87			

**Table 4:** Discriminant validity. (Fornell and Larcker [70].

	CSR	Employee trust	P-O fit	Employee loyalty
CSR	0.83			
Employee trust	0.77	0.87		
P-O fit	0.69	0.76	0.82	
Employee loyalty	0.72	0.73	0.76	0.85
Heterotrait–Monotrait. (HTMT)				
CSR	1			
Employee trust	0.79	1		
P-O fit	0.72	0.70	1	
Employee loyalty	0.74	0.68	0.72	1

### 4.3 Structural model

**Fig 3:**
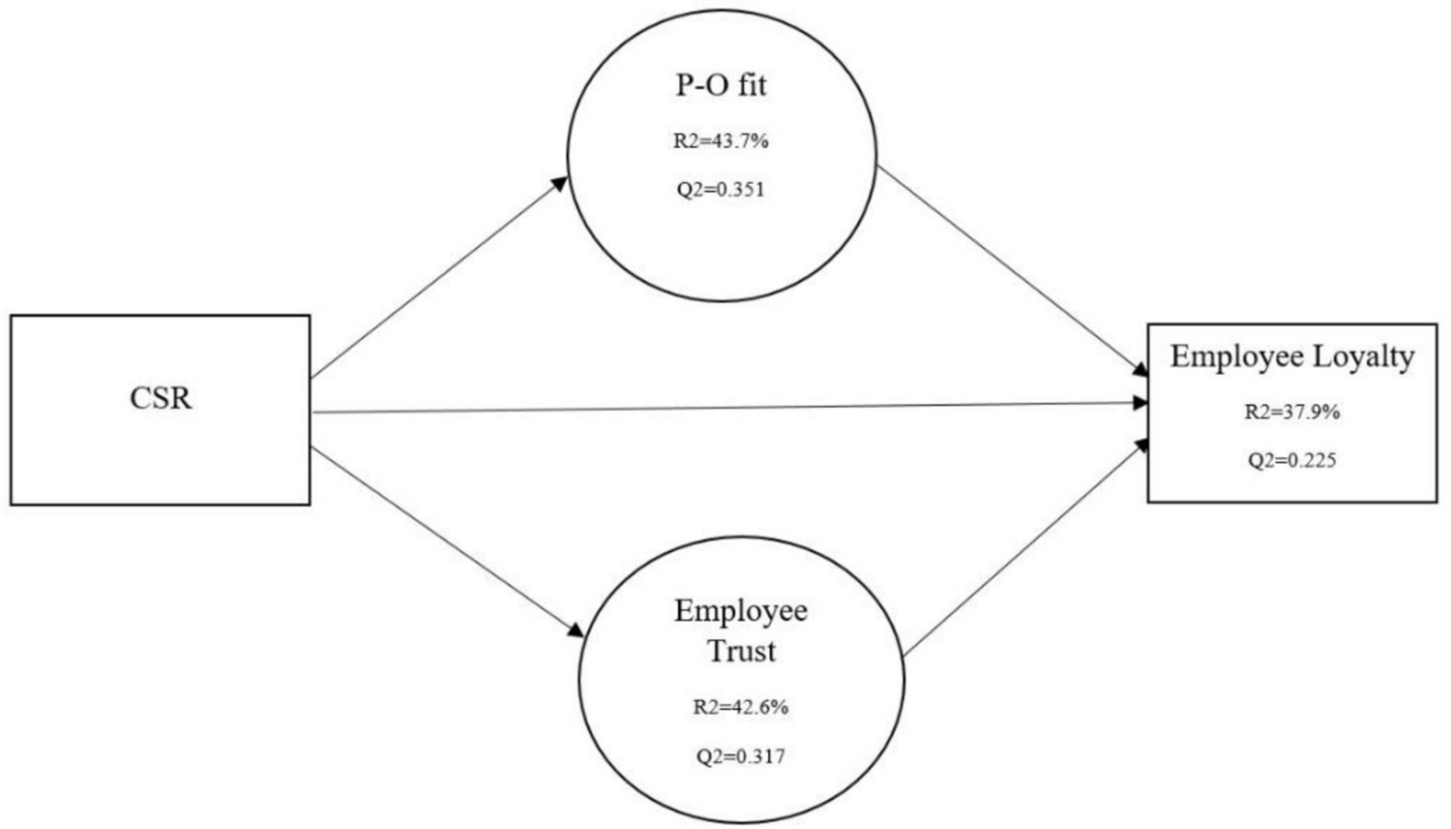
Structural model.

Hair et al. [71] recommended a bootstrapping approach with a sample of 5000 to examine the R^2^, beta value, predictive relevance Q^2^, and corresponding t-statistics in order to evaluate the structural model. The results show R^2^ values for P-O fit, employee trust and employee loyalty are 0.437, 0.425, and 0.379, respectively (See Fig 3). In addition, Q^2^ indicates a predictive relevance of the endogenous variable. A Q^2^ value greater than 0 shows predictive relevance in the model; however, a Q^2^ value below 0 indicates that the model is not predictively relevant. The results demonstrate the significance predictive relevance (See Fig 3). The results show that employee perception of corporate social responsibility has a significant positive impact on employee loyalty (β = 0.205, t = 2.41, p = 0.00), P-O fit (β = 0.464, t = 5.24, p = 0.00), and employee trust (β = 0.550, t = 6.08, p = 0.00), consequently, H1. H1a and H1b were accepted (see Table 5). Moreover, P-O fit significantly and positively influences employee loyalty (b = 0.527, t = 5.72, p < 0.01); thus, H2 was accepted. Similarly, the impact of employee trust shows a positive impact on their loyalty toward the organization (b = 0.419, t = 3.85, p < 0.01); therefore, H3 is also supported, as shown in Table 5. By running bootstrapping technique, a significant positive mediating impact of P-O fit in the relationship between CSR and employee loyalty exists (b = 0.244, t = 3.27, p < 0.00); likewise, employee trust also significantly mediates the link between CSR and employee loyalty (b = 0.230, t = 2.36 p > 0.01). Consequently, this study accepted H4, as shown in Table 5.

**Table 5:** Structural analysis and indirect effects.

Hypothesis	β	T-value	P-value	Decision	VIF
CSR-> Employee loyalty	0.205	2.41	0.00	Accepted	1.695
CSR-> P-O fit	0.464	5.24	0.00	Accepted	1.706
CSR-> Employee trust	0.550	6.08	0.00	Accepted	1.857
P-O fit-> Employee loyalty	0.527	5.72	0.01	Accepted	1.757
Employee trust-> Employee loyalty	0.419	3.85	0.01	Accepted	1.679
Indirect effect	β	T-value	P-value	Decision	
CSR-> P-O fit-> Employee loyalty	0.244	3.27	0.00	Accepted	
CSR-> Employee trust-> Employee loyalty	0.230	2.36	0.01	Accepted	

The mediating role was analyzed between the targeted variables. P-O fit shows a partial mediating impact between CSR and employee loyalty (b = 0.244, t = 3.27, p < 0.00). Moreover, a significant partial mediating effect of employee trust in the relationship between CSR and employee loyalty exists (b = 0.230, t = 2.36, p < 0.01). Hence, H4 is accepted (see Table 5).

### 5. Discussion

This study explored the influence of employee perception of corporate social responsibility on employee loyalty by direct and indirect variables such as P-O fit and employee trust.

Our study results explored that CSR has a significant positive impact on employee loyalty, indicating that companies that invest in CSR are more likely to have loyal employees. However, it is important to note that CSR activities significantly depend on the nature of the organizations. This result supports the findings of Park and Levy [61], Kim and Kim [72], Oh et al. [62], and Mao et al. [32] that organizations’ socially responsible activities for employees potentially enhance their awareness and professionalism toward sustainable development, success, and work commitment. Evidence from Afridi et al. [37] also validated that CSR activities not only enhance employee commitment but also contribute to innovative work behavior. Moreover, in the context of the tourism and hospitality industry, the results are aligned with the Latif et al. [3] study, which found the potential benefits of CSR initiatives in building customer loyalty in the hotel industry and suggests that stakeholders should consider incorporating socially responsible practices into their business strategies. The study results are consistent with social identity theory, suggesting that CSR initiatives develop employees’ positive work behavior, which directly affects work engagement and loyalty toward their organization [73].

The present study examined the mediation mechanism of P-O fit and employee trust between employee perception of CSR and loyalty. The mediating results were found significant, indicating that P-O fit display a partial mediating effect between CSR and employee loyalty. These findings are in line with the previous studies that have empirically shown the indirect impact of P-O fit in the relationship between CSR and employee commitment [41]. Evidence from Smith and Kumar [30] also validated the argument that when there is a high level of P-O fit, employees tend to perceive their organization’s CSR initiatives as more consistent with their personal values and beliefs, which enhances their commitment and loyalty. Similarly, employee trust also shows a partial mediating effect between CSR and employee loyalty. Previous research has examined the indirect role of trust in various organizational contexts. For instance, Iglesias et al. [74] found that trust partially mediates the relationship between CSR and customer loyalty in the hotel industry. Latif et al. [3] highlighted the mediating effect of trust on the relationship between CSR and employee performance in the educational sector. Moreover, Loor-Zambrano et al. [58] identified trust as a significant construct in the relationship between CSR and employee loyalty in the service industry. Therefore, our findings align with previous literature.

This study affirmed valuable insights into how CSR practices influence employee loyalty within the CPEC project setting. Moreover, this research adds to the broader field of CSR by highlighting the three major projects in a single framework study and elaborating on the importance of CSR and employee loyalty. The CPEC project, with its distinct features and challenges, offers a unique setting to explore how CSR initiatives can foster employee loyalty in a complex and dynamic environment. By filling this research gap, the findings offer valuable insights for practitioners and researchers interested in understanding and leveraging CSR practices to enhance employee psychological intentions within mega projects worldwide.

The mediating role of these two potential variables in project sectors has been ignored in the CSR literature. The results confirmed that P-O fit and employee trust factors must be taken into account in CSR initiatives in order to enhance employee loyalty. In light of stakeholder theory, results may be attributed to the fact that P-O fit is a core element that constitutes a number of positive work attitudes, including innovative work behavior, conflict management, and work commitment, which lead to organizational loyalty [4]. Similarly, Kim and Gatling [48] found employee trust as a consequence of CSR possesses a significant impact on work behavior. They reported employee trust as a significant antecedent of satisfaction.

### 5.1 Theoretical contribution

The findings of this study add empirical support to the existing literature on CSR in the area of project organization.

Although a number of studies have been conducted on the CSR-loyalty relationship in hotel industries, airport sectors, and other business industries, however, till now, no study has been done that explores CSR-employee loyalty in the context of the CPEC project. This study also attempts to explore P-O fit and employee trust role in the relationship between CSR-employee loyalty in the project sectors.

The outcome of this research underscores the need to construct and analyze a global conceptual model that considers the direct and indirect link between CSR-employee loyalty [3]. In the context of organizational sectors, P-O fit and employee trust are particularly relevant and should be considered in an advanced integrative model for further comprehensive elucidation of the CSR-employee loyalty link. In addition, this research is the very first to examine how CSR could improve employee loyalty in the project sectors. According to the results, the study explained that CSR initiatives could highlight the favorable work behavior of employees, and the organization can create a positive image and reputation. The outcomes of this research can help service firms to formulate strategies that are more socially responsible and reputable.

This study explains how P-O fit and employee trust affects the CSR-employee loyalty link. The perception of P-O fit and employee trust comprises cognitive and emotional components [75], and it serves as a basis for certain actions. Moreover, in the project sectors, P-O fit and employee trust may influence the CSR-loyalty relationship because of several factors (e.g., honesty, capability, and organizational support). The results are aligned with the prior studies [76], which emphasize the impact of P-O fit and employee trust in the context of CSR and employee work behavior. Nonetheless, these results are helpful to management scholars who are interested to understand and evaluate employee positive behavior through CSR activities.

### 5.2 Managerial implications

CSR approaches have gained substantial consideration in the management field, such as the tourism industry, services industry, and project sectors. Accordingly, employees expect organizations to involve in various CSR actions (e.g., employee well-being concerns, community involvement and welfare, sustainable environmental, ethical and social issues). Considering managerial implications and the competitive nature of the project sector, organizations must initiate CSR activities that might help the employee to display positive work behavior and reduce their turnout intentions. The research recommends that stakeholders understand the employees’ psychological behavior; precisely, the organization must carry out CSR activities for the well-being of the employees, which in turn, can lead to the strategic goal of the organization. In this paper, CSR has been found to be an efficient approach to managing organizational sustainability, which portrayed a significant impact on society because employee pays more attention to those organization that shows high value to their social problems [77]. Hence, CSR activities can be used as a strategic tool to manage employee emotions in order to perform better work tasks [72]. Findings indicate the significant implications of CSR activities for stakeholders who seek to establish high-level employee-organizational loyalty.

Moreover, developing CSR activities and creating favorable consequences are very important for project sectors. As CSR activities in organizations are linked with employees, which in turn, increases employees’ emotional and cognitive relationships. Stakeholders should conduct relevant CSR-related programs which frequently detect how CSR initiatives can affect employee work behavior and monitor whether organizational CSR activities can satisfy employees’ needs and expectations. By understanding the influence of CSR efforts, management can adjust and develop various CSR programs or strategies to meet employee expectations and increase their loyalty.

Many organizations have already acknowledged the role of P-O fit and employee trust in the formation of employee loyalty. However, they need to consider CSR activities to increase the relationship between organizations and employee loyalty. In order to enhance P-O fit and employee trust, stakeholders should develop adaptable CSR strategies that create a positive image among employees. Moreover, this is significantly relevant for developing countries such as Pakistan, where CSR novelty is still in the initial stages.

### 5.3 Limitations and future research direction

Like all research, the present study also had a few limitations that provide potential opportunities for further research. First, this study targeted employees from Pakistan working on CPEC projects operating in Pakistan. To explore the effect of corporate social responsibility on employee work behavior more comprehensively, other developing and developed countries can be considered for future research. Second, cross-sectional survey data were used to assess the study hypotheses. Future research could consider a longitudinal sample that could help determine the substantial change in CSR, P-O fit, employee trust, and employee loyalty that dynamically change. Moreover, further research could also determine the impact of other mediating variables between CSR and employee work behavior that possibly provide interesting outcomes. In addition, future research can also assess the impact of moderating variables such as role conflict, role ambiguity, organizational support, or cultural intelligence. Further research may also consider a large data sample derived from different business sectors such as the airport industry, SME sectors, or tourism industry.

## Supporting information

S1 Questionnaire (DOCX)

S2 Questionnaire (DOCX)
