# Correction: Impact of corporate social responsibility on employee loyalty: Mediating role of person-organization fit and employee trust

**DOI:** 10.1371/journal.pone.0320597

**Published:** 2025-03-17

**Authors:** Hebo Jin, Xuexiao Li, Guangsen Li

The following information is missing from the Funding statement: Shandong Province Higher Education Undergraduate Teaching Reform Research Project (M2022168) awarded to H.J., Shandong Province Education and Teaching Research Project (2023JXY010) awarded to H.J., and Teaching Reform Project of Qingdao University of Technology (F2023-039) awarded to H.J.
