# Supplementary material for: Impact of corporate social responsibility on employee loyalty: Mediating role of person-organization fit and employee trust
Source: PLoS One. 2024 Mar 21;19(3):e0300933. doi: 10.1371/journal.pone.0300933 (PMC10956873; doi:10.1371/journal.pone.0300933)
Supplement: S2 Questionnaire — (DOCX) [file pone.0300933.s002.docx]

Dear Participant,

This questionnaire is for research purposes only. Your responses are completely confidential and will be used solely for research purposes. Your data will not be identifiable in any findings. Your participation is voluntary, and your decision won't affect your relationship with the organization.

Thank you for your participation and valuable contribution.

(1= Strongly disagree, 1: Strongly Disagree; 2 Disagree; 3: Neither Agree nor Disagree; 4: Agree, 5: Strongly Agree)

Age:

Gender：

Region：

|  | **Corporate Social Responsibility** | **1** | **2** | **3** | **4** | **5** |
| --- | --- | --- | --- | --- | --- | --- |
| CSR1 | The company demonstrates commitment to ethical business practices |  |  |  |  |  |
| CSR2 | Our organization actively supports environmental sustainability initiatives. |  |  |  |  |  |
| CSR3 | Our company takes responsibility for the impact of its products/services on society |  |  |  |  |  |
| CSR4 | The company promotes social justice and equality. |  |  |  |  |  |
| CSR5 | Our company upholds high standards of integrity and ethics. |  |  |  |  |  |
| CSR6 | Employees perceive the company as being committed to ethical conduct in all aspects of business operations |  |  |  |  |  |
| CSR7 | The company invests in research and development to create environmentally friendly products/services. |  |  |  |  |  |
| CSR8 | Employees perceive the company as responsive to the needs and concerns of diverse stakeholders. |  |  |  |  |  |
|  | **Person-organization fit** |  |  |  |  |  |
| P-O fit1 | I feel that my skills and abilities are a good match for the needs of this organization. |  |  |  |  |  |
| P-O fit2 | The culture of this organization aligns well with my working style and preferences. |  |  |  |  |  |
| P-O fit3 | I feel a sense of belonging and comfort in the work environment of this organization. |  |  |  |  |  |
| P-O fit4 | I feel that my personal values align closely with the values promoted by the organization. |  |  |  |  |  |
| P-O fit5 | I feel a sense of harmony between my personal work ethics and the organization's ethical standards. |  |  |  |  |  |
| P-O fit6 | The level of innovation and risk-taking in this organization aligns with my approach to work. |  |  |  |  |  |
|  | **Employee Trust** |  |  |  |  |  |
| ET1 | I trust the leadership team of this organization to make decisions that benefit employees. |  |  |  |  |  |
| ET2 | I have confidence in the company's commitment to fulfilling its promises to employees. |  |  |  |  |  |
| ET3 | I trust that the organization values and respects the opinions of its employees. |  |  |  |  |  |
| ET4 | I trust that the company prioritizes employee well-being and safety. |  |  |  |  |  |
|  | **Employee Loyalty** |  |  |  |  |  |
| EL1 | I feel a strong sense of commitment to this organization. |  |  |  |  |  |
| EL2 | I am willing to put in extra effort to help this organization succeed. |  |  |  |  |  |
| EL3 | I see a long-term future for myself at this company. |  |  |  |  |  |
| EL4 | I have a strong emotional connection to this organization. |  |  |  |  |  |
| EL5 | I feel a deep attachment to the values and culture of this organization. |  |  |  |  |  |
| EL6 | I rarely think about looking for a job at another company. |  |  |  |  |  |
